# The efficacy of infrared diode laser in enhancing the regenerative potential of human periodontal ligament stem cells (hPDLSCs)

**DOI:** 10.1186/s12903-024-05038-3

**Published:** 2024-10-29

**Authors:** Mohamed M. Abo El-Dahab, Ghada Nour El Deen, Mahmoud Shalash, Mostafa Gheith, Ahmed Abbass, Riham M. Aly

**Affiliations:** 1 Department of Basic Dental Science, Oral and Dental Research Institute , National Research Centre, Dokki, Giza, Egypt; 2 Stem Cell Laboratory, Center of Excellence for Advanced Sciences, National Research Centre, Dokki, Giza, Egypt; 3 Molecular Genetics & Enzymology Department, Human Genetic & Genome Research Institute, National Research Centre, Dokki, Giza Egypt; 4 Surgery and Oral medicine Department, Oral and Dental Research Institute, National Research Centre, Dokki, Giza, Egypt; 5 National Institute of Laser Enhanced Sciences, Cairo University, Giza, Egypt

**Keywords:** hPDLSCs, Infra-red diode laser, Stemness, Osteogenic differentiation, Photobiomodulation

## Abstract

**Background:**

The present study aimed to investigate the effects of infrared diode laser irradiation on the proliferation and differentiation capacity of periodontal ligament stem cells (hPDLSCs), which are optimal cell sources for periodontal regeneration.

**Methods:**

hPDLSCs were isolated and characterized by flow cytometric analysis of mesenchymal stem cell markers, and their trilineage differentiation capacity was tested. hPDLSCs were then cultured and irradiated with infrared diode laser (970 nm) at a power of 200 mW and a fluence of 4 J/cm^2^ for 3 s. MTT assay was performed to assess cellular proliferation. Cell cycle analysis was performed, and the impact of infrared diode laser irradiation on the stemness and osteogenic differentiation potential of hPDLSCs was evaluated via RT‒PCR.

**Results:**

Infrared diode laser application enhanced the stemness, viability, proliferation, and differentiation of PDLSCs. Stem cell markers (*OCT*4, *SOX*2, and *NANOG*) were significantly upregulated in hPDLSCs exposed to laser irradiation. There was significant overexpression of *RUNX2*,* ALP*,* OPN*,* and OCN* on day 14 after laser application.

**Conclusions:**

These findings provide valuable insights into the specific applications of infrared diode lasers to effectively regenerate periodontal tissues. The results can aid in the development of precise clinical protocols aimed at enhancing osseointegration and promoting tissue regeneration. Ultimately, the combination of infrared diode laser with hPDLSCs is promising for stimulating periodontal regeneration.

## Introduction

The prevalence of periodontal disease in the adult Egyptian population ranges from 69.4 to 89.8% [1]. Periodontal disease in most patients is usually associated with different degrees of alveolar bone resorption. Today, the main therapeutic approaches include nonsurgical periodontal treatment, surgical treatment and the use of bone replacement materials for repair. Conventional nonsurgical periodontal therapy for periodontal disease could control periodontal inflammation and prevent the progression of the disease; however, it cannot repair the destroyed alveolar bone. Although surgical treatment and the use of bone replacement materials can partially enhance alveolar bone, the latter approach is restricted by various factors, and its long-term outcomes remain uncertain.

A number of therapeutic procedures, including osseous grafting and guided tissue regeneration, have been developed to improve regeneration of the periodontium. However, to accomplish successful periodontal regeneration, regenerated tissues must restore tissues that were previously destroyed.

Periodontal ligament stem cells (PDLSCs), obtained from periodontal ligaments with multilineage differentiation abilities, are considered promising candidate stem cells for periodontal regeneration. Previous research have demonstrated that PDLSCs can differentiate into cementoblasts and osteoblasts and subsequently generate periodontium-like tissues [2, 3].

Low-level laser therapy (LLLT), also referred to as photobiomodulation is currently often employed as a clinical technique for accelerating various treatment procedures. LLLT has been proven to enhance the rate of cell proliferation in different kinds of cells, including stem cells [3–7]. Photobiomodulation directly affects cellular function by activating different intracellular signaling pathways, leading to increased expression of growth factors and cytokines. Moreover, the modulation of MAPK and PI3K/Akt pathways through laser stimulation affects gene expression, cell cycle progression, and differentiation. Numerous explanations of the mechanism by which laser photobiomodulation operates have been documented. One hypothesis proposes that laser selectively captures mitochondrial energy, therefore stimulating the respiratory chain to generate additional ATP and initiate mitosis by augmenting the synthesis of RNA [8]. An alternative theory suggests that photobiomodulation (PBM) induces the production of reactive oxygen species (ROS), which subsequently triggers the activation of intrinsic growth factors that are responsible for the growth and differentiation of stem cells [9]. Consequently, laser irradiation enhances cellular metabolism and viability by promoting the production of reactive oxygen species (ROS) and mitochondrial absorption [10].

The versatility and efficacy of infrared diode lasers, which operate at wavelengths of 810 nm, 940 nm, and 980 nm, have revolutionized dental treatments [11]. In comparison to conventional low-level laser therapy (LLLT), these lasers provide numerous benefits. Initially, their increased power output enables them to be used in a wider variety of applications, rendering them effective for both soft and hard tissue procedures [12]. The deeper tissue penetration that their extended wavelengths provide is especially advantageous within the oral cavity, such as in endodontics and periodontal therapy. Infrared diode lasers are also employed in soft tissue surgery for procedures such as gingivectomy and frenectomy where precise cutting with minimal hemorrhage and discomfort is achieved [13]. They are also effective in periodontal therapy, where they aid in pocket decontamination, thereby fostering healing and reducing inflammation. In endodontics infrared diode laser is often used to disinfect root canals by deeply penetrating the dentinal tubules to combat bacteria [14]. Furthermore, they are very useful for pain management by means of photobiomodulation therapy, which facilitates speedier healing and reduces inflammation and pain [15]. The versatility of these lasers is further enhanced by their capacity to operate in both continuous wave and pulse modes which enables the development of customized treatment protocols that optimize patient outcomes and reduce the impact on adjacent tissues [16].

Although, photobiomodulation therapy has been shown to have beneficial effects on dental derived stem cells, the existing scientific literature does not offer a conclusive decision on the optimal parameters to promote cell viability, proliferation, and differentiation [17]. While several previous research have described positive impact of laser irradiation on cells from various sources [4, 18–21], there are also studies suggesting that it has little impact on cell proliferation and differentiation or may even lead to a decline in such characteristics [22].

As a result of this, it has proven challenging to establish accurate conclusions and guidelines for applications both in laboratory and clinical settings. Thus, additional well-planned investigations are required to determine the optimal settings for each specific wavelength and cell type.

Infrared diode laser is considered an ideal wavelength for possible applications in periodontal regeneration, either in vitro or in a clinical environment, due to its advantageous ability to penetrate oral tissues deeply. However, this topic has not been thoroughly investigated. We hypothesized that the regenerative capacity of hPDLSCs would be enhanced upon exposure to infrared diode laser. Thus, the present study aimed to investigate the effects of infrared diode laser irradiation on the proliferative capacity and differentiation potential of periodontal ligament stem cells, which are optimal cell sources for periodontal regeneration. Our results demonstrated that infrared diode laser irradiation can enhance the stemness, viability, proliferation, and differentiation capacity of hPDLSCs.

## Materials & methods

### Isolation of human periodontal ligament stem cells

Human impacted third molars (*n* = 3) with no signs of infection indicated for extraction were used in this study. Informed consent was obtained from the donors. All experimental procedures were approved by the Ethical Committee of the Medical Research of the National Research Centre, Egypt. After extraction, the molars were immediately placed in sterile polypropylene tubes with a volume of 50 ml containing Dulbecco’s modified Eagle’s medium (DMEM) (Gibco, USA) supplemented with 100 units/mL of penicillin and 100 µg/mL of streptomycin (Thermo Fisher Scientific, USA). The periodontal ligament tissue was scraped off the root surface using a sterile lancet and washed five times with phosphate buffered solution (PBS) containing penicillin and streptomycin. The periodontal ligament fragments were combined and finely cut and then digested with 2 mg/mL collagenase type I (Serva Electrophores, Germany) for approximately 20 min at room temperature in a water bath shaker. Single cells were seeded into culture plates with DMEM supplemented with 15% fetal bovine serum (Lonza, Basel, Switzerland), 10,000 U/ml penicillin, and 10,000 ng/ml streptomycin (Thermo Fisher Scientific, USA). After that, the plates were incubated in 5% CO2 at 37 °C. Passaging was performed at approximately 70% confluency using TrypleSelect^®^ (Thermo Fisher Scientific, USA).

### Characterization of isolated cells by flow cytometry analysis

Flow cytometry analysis was conducted to confirm the presence of mesenchymal stem cells (MSCs) in the cultured cells. This study specifically targeted MSC-specific markers (CD73 and CD44) and a hematopoietic marker (CD34). The PE-conjugated CD73 and CD44 antibodies were acquired from R&D Systems (UK) [23]. The cells were exposed to an antibody specific to each surface marker for a duration of 20 min at a temperature of 25 °C. Subsequently, the cells were analyzed using flow cytometry (Beckman Coulter Elite, USA).

### In vitro multilineage differentiation analysis

The multilineage differentiation potential of hPDLSCs was evaluated by using lineage-specific staining. After resuspension in culture medium, 5,000 cells were plated in 12-well culture plates. Upon adherence to the plates, the medium was changed to osteogenic, adipogenic, or chondrogenic differentiation medium, and the cells were cultured for a 3-week period before their ability to efficiently differentiate was assessed. Oil Red O (0.3% solution) (Sigma-Aldrich, Germany) was used to stain hPDLSCs to detect the presence of extracellular lipid droplets. For chondrogenic differentiation, sulfated glucose amino glycans were detected by Alcian blue staining (Sigma-Aldrich, Germany). To assess whether hPDLSCs can differentiate into osteogenic lineages, osteoblast-like cells were stained with 1% Alizarin Red S solution (Sigma-Aldrich, Germany).

### Laser application protocol

hPDLSCs were allocated into an experimental group, which received laser irradiation, and a control group, which did not receive laser irradiation. Infra-red Diode laser (Sirona Dental Laser System, Bensheim, Germany) at 940 nm wavelength at a power of 200 mW and a fluence of 4 J/cm^2^ for 3 s was used in this study [20, 24]. Application was conducted at a height of 0.5 cm to each cell seeded well through the fiber optic (SiroLaser fibers 320) in continuous circular motion movement in noncontact mode. The intervals and total number of irradiations were determined by the experimental design under investigation.

For cell proliferation assessment, the cells were seeded in 96-well plates at a density of 1 × 10^4^ cells/well and were allowed to attach overnight. Laser was applied 24 h following cellular attachment in a single dose directly perpendicular to each tested well using the above-mentioned parameters. Cellular viability and proliferation were assessed at days 7 & 14 days after irradiation via MTT assay. For osteogenic differentiation experiments, cells were seeded in 12 well plates at a density of 1.0 × 10^4^ cells/cm^2^ and infrared diode laser was applied to hPDLSCs with every change of medium during the entire induction period with the above-mentioned parameters and following our previously described protocol [21]. Uniform and consistent movements were applied across the full surface of the culture plates.

### Assessment of cell proliferation via 3-[4,5-dimethylthiazol-2-yl]-2,5 diphenyl tetrazolium bromide (MTT assay)

To determine whether laser exposure on days 7 and 14 affected the viability of the cells, MTT assessment was performed as described previously [20, 25, 26]. The cells were washed with phosphate buffered solution and then placed in an incubator for two hours in a 5% CO2 incubator at 37 °C with MTT solution (1 mg/ml). Subsequently, dimethyl sulfoxide (POCH, Poland) was added to dissolve the formazan crystals after the supernatant was discarded. Spectrophotometric analysis was conducted to determine the absorbance at two reference and test characteristic wavelengths (570 nm and 630 nm, respectively) using a VarioScan LUX plate reader (Thermo Fisher Scientific, USA). Cells that were not exposed served as a control.

### Cell cycle analysis

Following exposure to infrared diode laser irradiation, human periodontal ligament stem cells (hPDLSCs) were placed at a density of approximately 1.19 × 10^3^ cells/cm^2^ and subsequently harvested after 2 days. Subsequently, a solution of 70% ethanol was introduced to the cells for a duration of 12 h. Following PBS washing, a total of 2 × 10^5^ cells were stained with 200 µl of propidium iodide for 30 min at 37 °C under dark conditions. The samples were analyzed using a Cytomics FC500 flow cytometer (Beckman Coulter, USA) and compared to cells that were not exposed to laser irradiation.

### Induction of osteogenic differentiation

Osteogenic differentiation for hPDLSCs seeded at 1.0 × 10^4^ cells/cm^2^ density was initiated by replacing the culture media with osteogenic induction media containing DMEM, 10% fetal bovine serum 5 mM β-glycero phosphate, 100 µM L-ascorbic acid 2-phosphate, 0.01 µM dexamethasone, 2 mM L-glutamine, for a duration of 14 days. The medium was replaced three times a week. The cells were subjected to laser irradiation, following our previously described protocol [24] where infrared diode laser was applied to hPDLSCs with every change of medium during the entire induction period at a power of 200 mW and a fluence of 4 J/cm^2^ for 3 s with uniform movement over the entire surface of the culture plates. The results were compared to those of the control unirradiated hPDLSCs.

### Alizarin red staining

Alizarin red staining was employed to verify the successful differentiation of hPDLSCs as evidenced by in vitro mineralization at 7 and 14 days. hPDLSCs were fixed for 45 min with 4% paraformaldehyde (Sigma‒Aldrich, USA) and rinsed with double distilled water. Then, 1% Alizarin Red S (Sigma‒Aldrich, USA) was added for 20 min. The mineralized deposits were examined using an inverted light microscope (Leica, Germany).

### Quantitative real-time PCR analysis

For relative gene expression of stemness markers with and without laser exposure, total RNA of MSCs, cultured in basal medium, was isolated to evaluate laser effect on the stemness markers. As for osteogenic differentiation potential of hPDLSCs subjected to laser irradiation, total RNA of was also extracted on day 14 after induction to assess the effect of laser beam on osteogenic differentiation potential. Total RNA extraction was done using TRIZOL Reagent (Invitrogen Life Technologies, NY, USA). The concentration of extracted RNA was quantified by spectrophotometer (GeneQuant Pro; Amersham Biosciences, USA) at wavelengths of 260 and 280 nm. Total RNA was reversely transcribed into first-strand cDNA by using the *COSMO* cDNA PLUS Synthesis Kit. The first-strand cDNA products were used as templates. The expressions of stemness markers and osteogenic genes were determined by SYBR green-based real-time PCR analysis using HERA qPCR SYBR^®^ Green master mix. The sample was analyzed using the LightCycler^®^ 480 Multicolor Real-Time Detection System (Roche). POU class 5 homeobox (*Oct4*), SRY-box transcription factor 2 (*Sox2*) and Nanog homeobox (*Nanog*) were used as undifferentiated stem cell markers. For bone- related markers, we examined mRNA expressions of runt-related transcription factor 2 (*Runx2*), alkaline phosphatase (*ALP*), osteopontin (*OPN*) and osteocalcin (*OCN*). All primer sequences are displayed in Table 1. The PCR was performed as follows: denatured at 95 °C for 2 min at the beginning, then denatured at 95 °C for 5s, annealing at 56 ~ 58 °C for 30s, and extending at 72 °C for 20s, for 30 cycles. Gene expression was normalized by the expression of housekeeping gene glyceraldehyde-3-phosphate dehydrogenase (*GAPDH*) in the same sample. The data represented the average fold changes with standard deviation. All experiments were carried out in triplicate.

**Table 1:** Sequences of primers used for rt- PCR

Gene	Primer sequence
*GAPDH*	Forward: 5′-TGTGTCCGTCGTGGATCTGA-3′ Reverse: 5′-TTGCTGTTGAAGTCGCAGGAG-3′.
*OCT4*	Forward: 5′-CCCGAAAGAGAAAGCGAAC-3′ Reverse: 5′-CACTCGGACCACATCCTTCT-3′
*SOX2*	Forward: 5′-GCCGAGTGGAAACTTTTGTC − 3′ Reverse: 5′- GGGCAGCGTGTACTTATCCTT-3′
*NANOG*	Forward: 5′-GTCCCGGTCAAGAAACAGAA − 3′ Reverse: 5′- GCTGAGGTTCAGGATGTTGG-3′
*RUNX2*	Forward: 5′- CCGTCCATCCACTCTACCAC-3′ Reverse: 5′- CCAGAGGCAGAAGTCAGAGG-3′
*ALP*	Forward: 5′- GGAGATGGGATGGGTGTCT-3′ Reverse: 5′- CCACGAAGGGGAACTTGTC-3′
*OPN*	Forward: 5′- GCCGAGGTGATAGTGTGGTT − 3′ Reverse: 5′- GCTTTCCATGTGTGAGGTGAT-3′
*OCN*	Forward: 5′-CCCTCTCTCTGCTCACTCTGCT-3′ Reverse: 5′-TGTGTTGTCCCTTCCCCTCT-3′

### Statistical analysis

Statistical analysis was performed using SPSS Statistical version 20 (SPSS, USA). Results were expressed as mean ± SD. For analysis of proliferation, the normality of the distribution parameters was evaluated by the one-sample Kolmogorov‒Smirnov test first; then, for normal distribution, one-way ANOVA and post hoc tests were used to assess differences between groups. For RT‒PCR analysis, data were evaluated using one- way ANOVA test and corrected by Bonferroni post-hoc test. P values were described in figures and P ˂ 0.05 was considered as statistically significant.

## Results

### Successful isolation of human periodontal ligament stem cells (hPDLSCs)

**Fig. 1:**
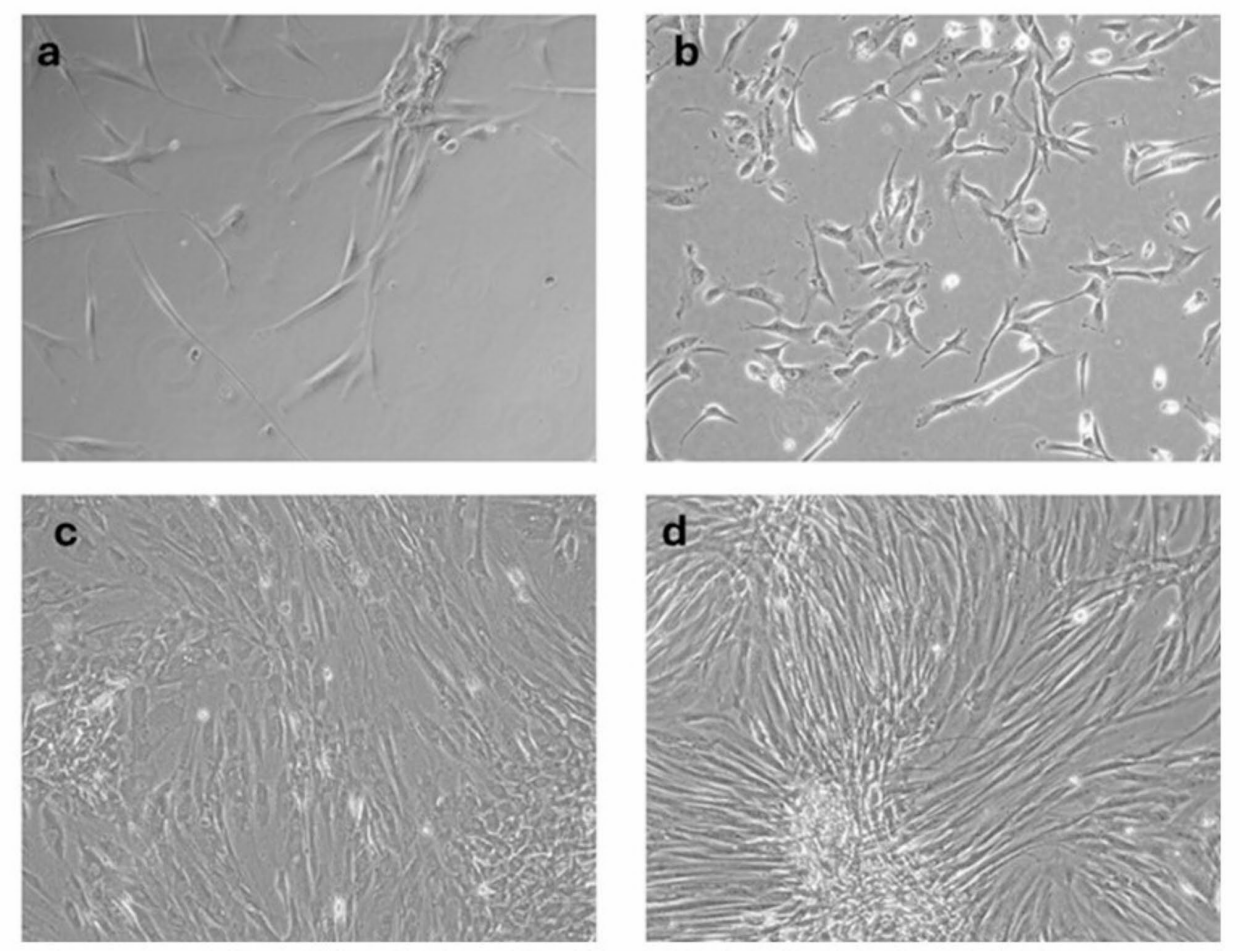
Morphological appearance of isolated human periodontal ligament stem cells derived three days after seeding. Spindle-shaped cells attached to the culture plate **(a)** followed by colony formation **(b)** by day five. The colonies increased in size **(c)** until they reached 70 to 80% confluence by day 10 **(d)**. (Magnification 10×)

Periodontal ligament stem cells (hPDLSCs) were effectively isolated from the scraped periodontal ligaments (Fig. 1). Individual fibroblast-like cells began to appear within 3 days following isolation. The cellular growth exhibited a typical pattern of stem cell proliferation, characterized by initial focal cellular attachment, followed by the formation of colonies around day 5. The colonies grew and merged until they reached a confluence of 70% by days 7 to 10.

### Isolated hPDLSCs exhibit mesenchymal stem cell phenotypic attributes

**Fig. 2:**
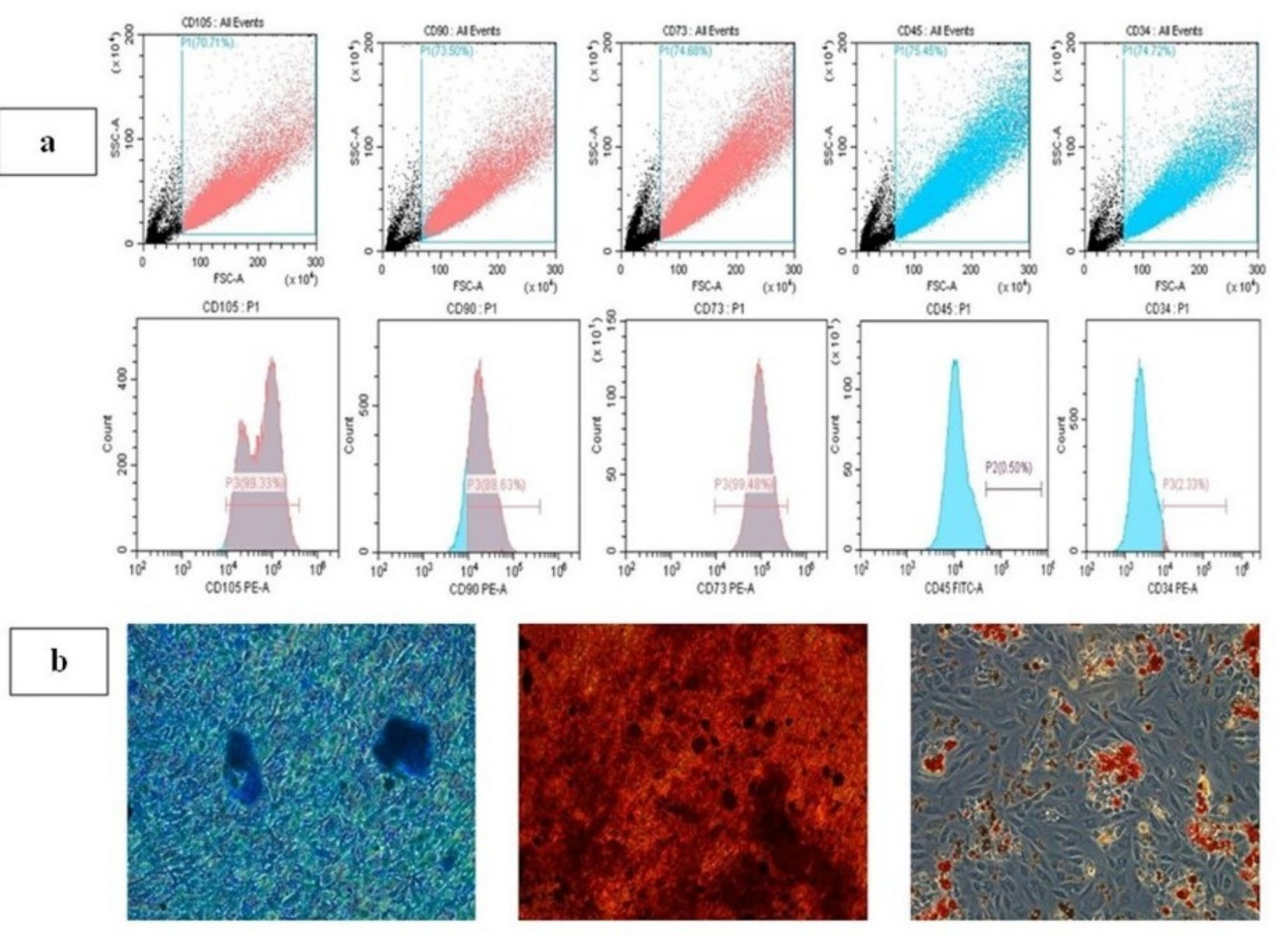
Characterization of isolated cells by flow cytometry (FACS) and trilineage differentiation. **(a)** Flow cytometry histograms illustrating the phenotypic profile of hPDLSCs, revealing positive expression of CD105, CD90 and CD73 and negative expression of CD45 and CD34. Osteogenic, adipogenic and chondrogenic differentiation of hPDLSCs **(b)**. hPDLSCs were stained with Alcian blue (left), Alizarin Red solution (middle), or Oil Red O (right) after 21 days of induction. (Magnification 10×)

The mesenchymal stem cell identity of the isolated cells was confirmed by immunophenotypic analysis via FACS, which revealed that the isolated cells were positive for the mesenchymal stem cell markers CD73 (99.48), CD90 (88.63%), and CD105 (99.3%). Additionally, the cells exhibited minimal expression of the hematopoietic stem cell markers CD34 (2.33%) and CD45 (0.50%) (Fig. 2a). The stem cell identity was further confirmed by successful trilineage differentiation (Fig. 2b). After 21 days of induction in suitable differentiation media, osteogenic, adipogenic, and chondrogenic lineages were confirmed with suitable dyes. Orange‒red calcium deposits were observed and indicated osteogenic differentiation. Additionally, adipogenic differentiation was identified by the presence of red-stained lipid droplets, and chondrogenic differentiation was identified through positive staining with Alcian blue solution indicating the presence of glycosaminoglycans.

### Infrared diode laser irradiation promotes cell viability and proliferation

**Fig. 3:**
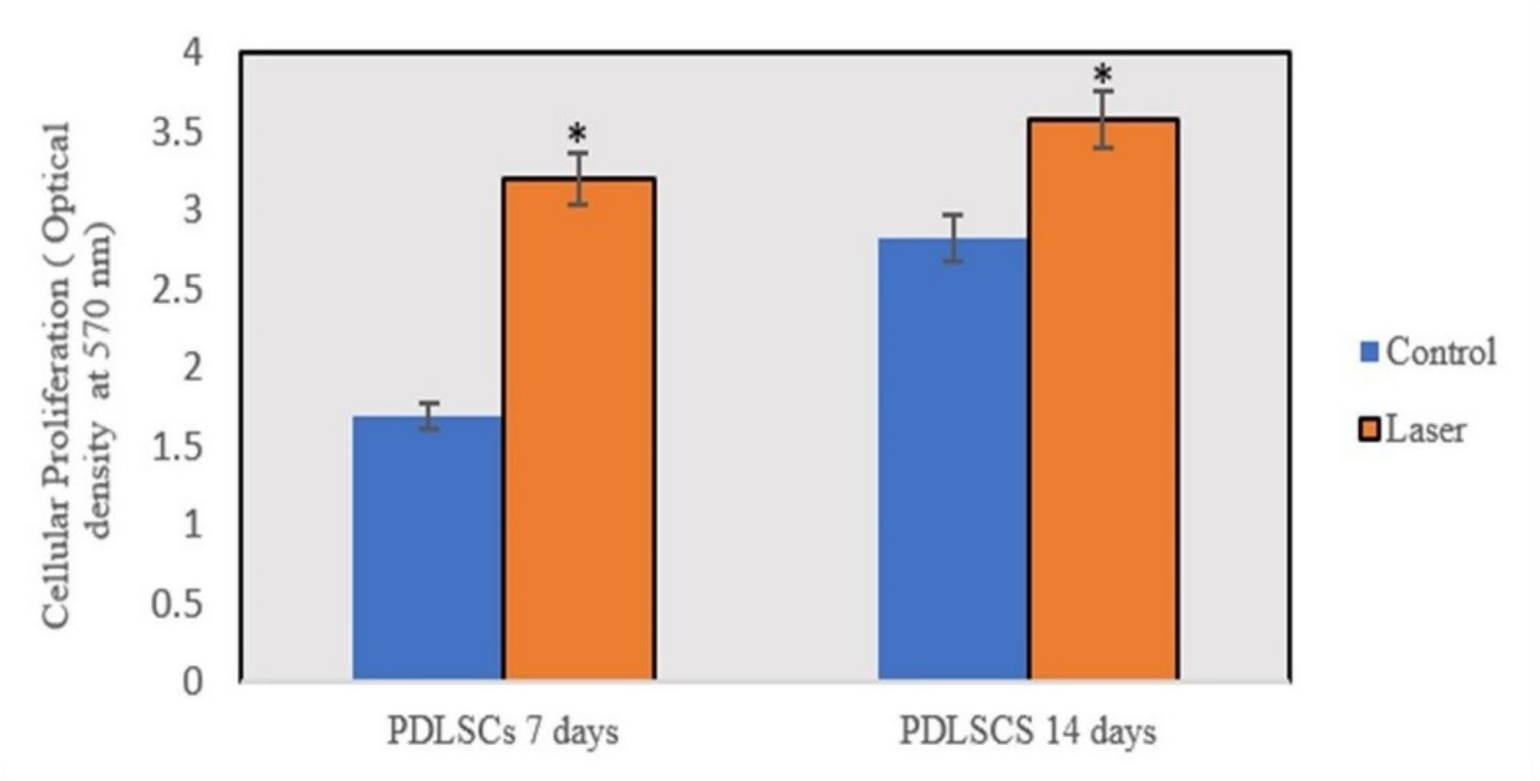
MTT cell proliferation assay of hPDLSCs. Significant differences were illustrated between hPDLSCs subjected to laser and the control on days 7 and 14 (* *p* < 0.05). The bars represent the mean ± standard error

The proliferation of hPDLSCs was significantly greater in cells exposed to the laser on both day 7 (*p* = 0.004) and day 14 than in cells that were not irradiated (Fig. 3). Moreover, the rate of proliferation was lower in the control group than in the irradiated hPDLSCs (*p* = 0.01). The results are presented as the means ± SDs.

### hPDLSCs cell cycle analysis after infrared diode laser irradiation

**Fig. 4:**
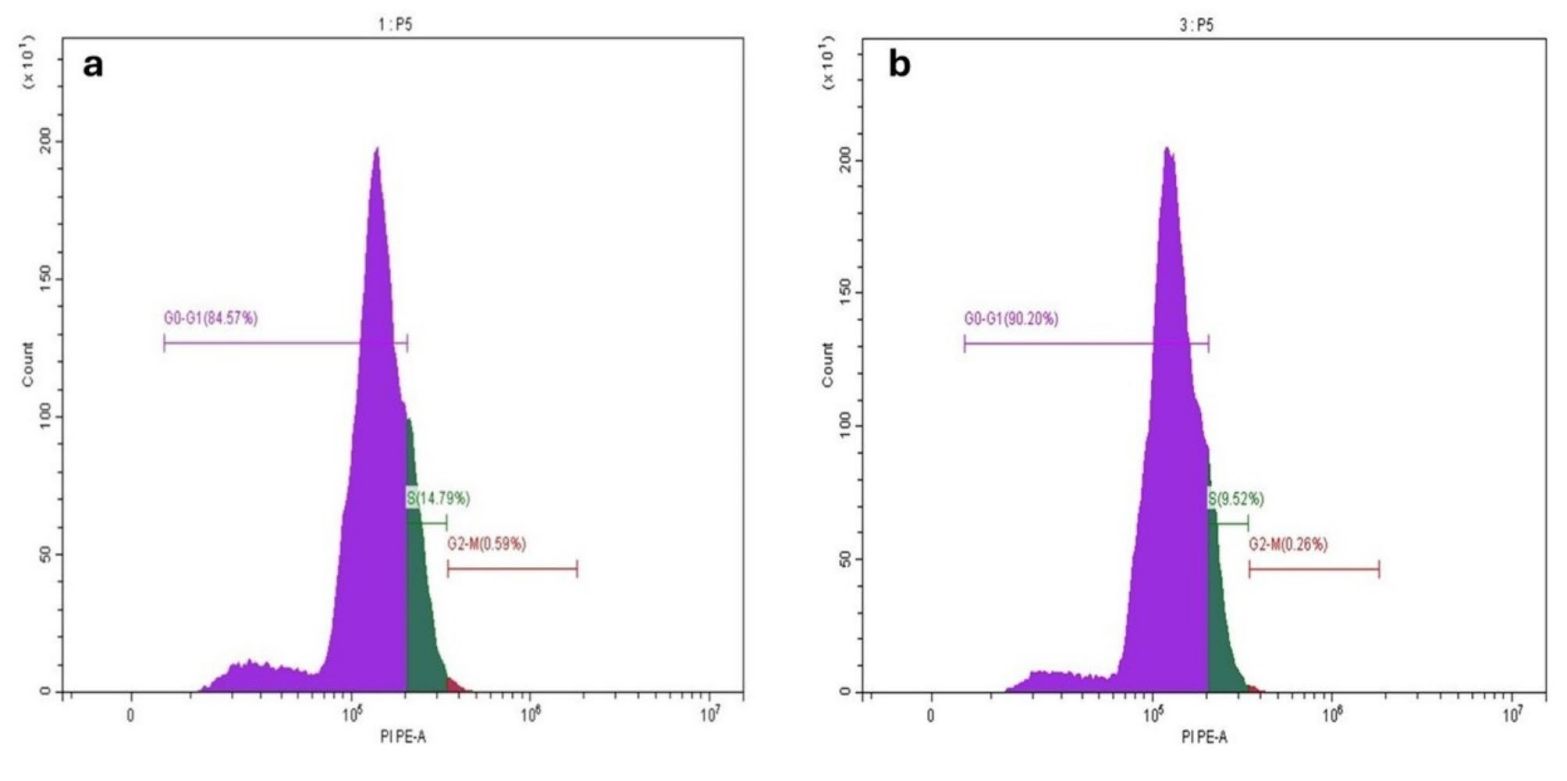
Cell cycle analysis by flow cytometry. The percentage of irradiated hPDLSCs **(a)**, in the S phase increased and decreased in G1 phase compared to unirradiated hPDLSCs **(b)**

Cell cycle analysis revealed that infrared diode laser irradiation enhanced the percentage of hPDLSCs in the S phase and reduced the percentage of hPDLSCs in the G1 phase in comparison to the control (Fig. 4).

### Infrared diode laser irradiation enhances stemness-related gene expression

**Fig. 5:**
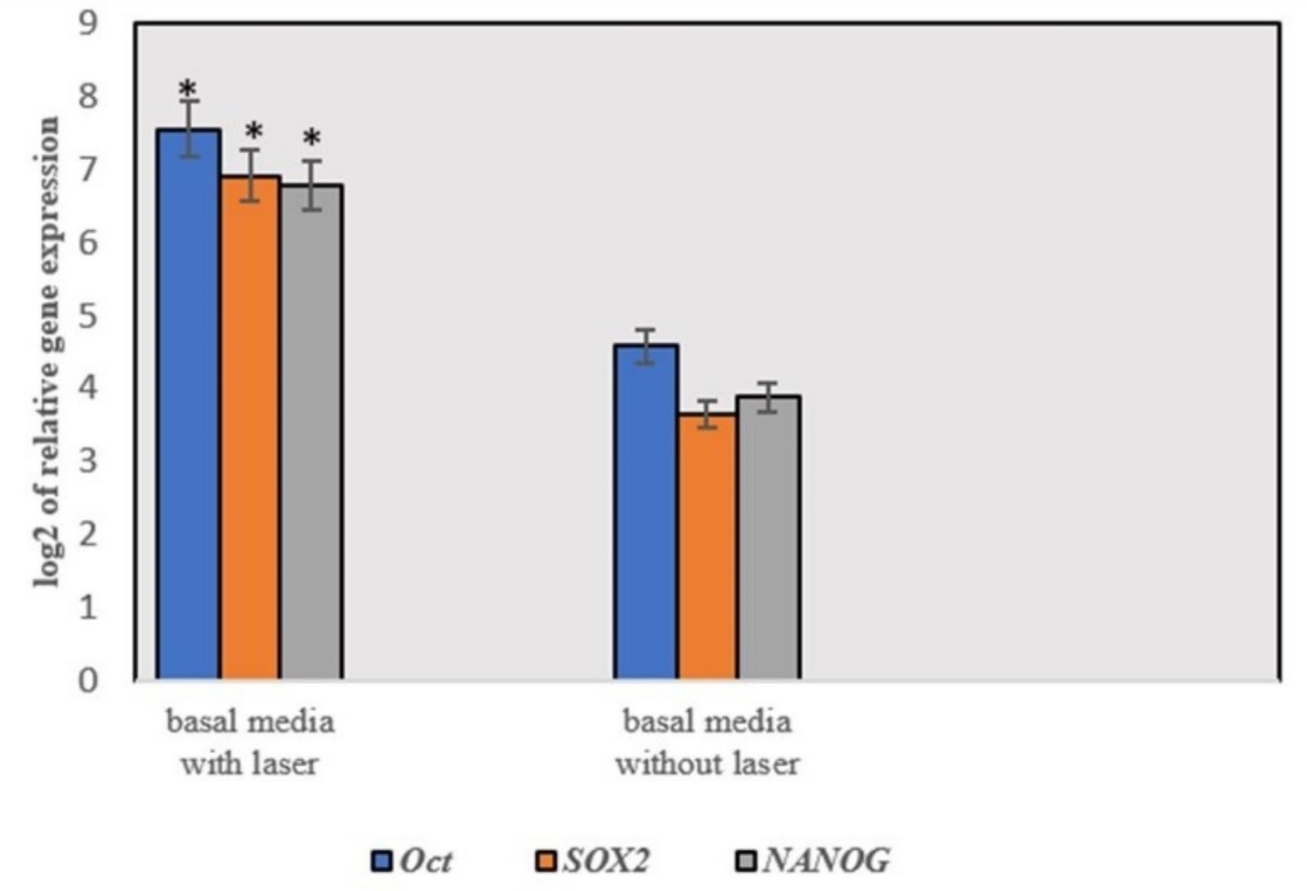
Relative mRNA expression of stemness markers (*OCT*4, *SOX*2, and *NANOG* ) in hPDLSCs exposed to laser in relation to unexposed cells. All mRNA expression levels were normalized to the GAPDH expression level. (*p < 0.05)

The expression of stem cell markers (*OCT4*,* SOX2*, and *NANOG*) was significantly higher in hPDLSCs after exposure to infrared diode laser irradiation than in unexposed cells cultured in basal media (Fig. 5).

### Infrared diode laser treatment promotes osteogenic differentiation of hPDLSCs

**Fig. 6:**
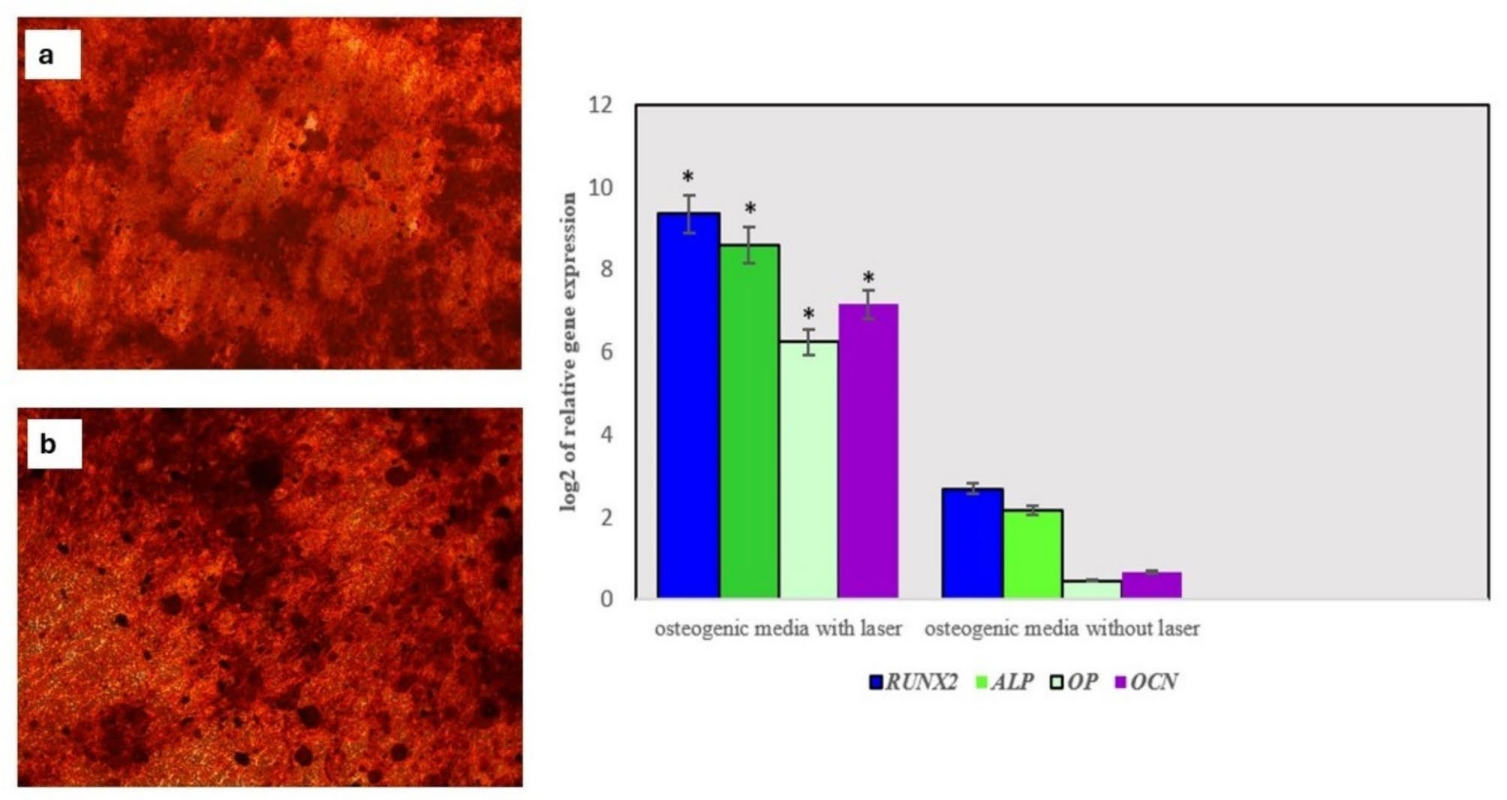
The osteogenic differentiation potential of hPDLSCs under infrared diode laser irradiation. Alizarin red staining at 14 days (left) in control hPDLSCs **(a)** and irradiated cells **(b)** (10X magnification). Relative expression of osteogenic markers in laser-exposed hPDLSCs cultured in osteogenic media compared with unexposed cells (right). The values were corrected using *GAPDH* expression as a reference gene. * indicates statistical significance at *P* < 0.05

Multiple mineralized nodules were detected by Alizarin red staining in hPDLSCs after the induction of osteogenic differentiation. Nevertheless, macroscopic assessment revealed increased nodule formation in hPDLSCs subjected to laser irradiation. This observation was further confirmed by RT‒PCR **(**Fig. 6**)**. In unirradiated hPDLSCs, the *RUNX2*,* ALP*,* OPN*, and *OCN* genes, which are responsible for bone formation and mineralization, were significantly overexpressed in hPDLSCs cultured in osteogenic media after laser exposure. This overexpression occurred on day 14 after laser application and was normalized to the *GAPDH* reference gene (*p* < 0.05).

## Discussion

Restoring damaged oral and craniofacial tissues represents a significant challenge in addressing diverse dental problems. Human periodontal ligament stem cells can regenerate alveolar bone and maintain the homeostasis of periodontal tissues [27–29]. Furthermore, hPDLSCs show promise for regenerating tissues, including cementum, which plays a vital role in the restoration of periodontal attachment and overall oral health. Despite the potential of hPDLSCs for tissue regeneration, several obstacles remain to be addressed. These include directing hPDLSCs toward specific cell lineages, including cementoblasts and osteoblasts, increasing the rate of cell growth, and enhancing the ability of the cells to differentiate into osteogenic lineages. Therefore, it is crucial to optimize differentiation methods to achieve tissue-specific regeneration. Low-level laser irradiation has been extensively studied and shown to have a beneficial effect on biological processes, such as the proliferation and differentiation of different types of cells [2, 3, 5, 6]. However, there has been limited research examining the influence of low-level laser irradiation, specifically infrared diode lasers, on periodontal ligament stem cells, which are crucial for regeneration of the whole periodontium. Thus, our study focused on examining the impact of infrared diode laser irradiation as an enhancer of the regenerative potential of hPDLSCs in terms of proliferation and osteogenic differentiation potential.

Despite comprehensive studies investigating the impact of laser irradiation on cells from different sources, the use of various laser parameters and cell types has resulted in difficulties in making accurate comparisons. The results of our investigation demonstrated a significant increase in the proliferation of hPDLSCs compared to control cells on days 1 and 7 after irradiation with infrared diode laser (970 nm) at a power of 200 mW and a fluence of 4 J/cm^2^. These outcomes are similar in a way with those of similar relevant studies on the effect of laser irradiation on other sources of mesenchymal stem cells on viability/proliferation. Alhazmi et al., assessed the cytocompatibility and differentiation capacity of gingival derived stem cells following laser irradiation at a wavelength of 980 nm, at two different energy densities of 1.5 J and 3 J, for a duration of 60 s. Their results demonstrated a notable enhancement in cell viability, suggesting that low level laser irradiation was both non-toxic to gingival derived stem cells and promoted cellular proliferation [30]. Turrioni et al. investigated the effects of different energy fluences of infrared light source (850 nm, 40 mW/cm^2^) [31]. The fluences of 4 J/cm^2^, 8 J/cm^2^, and 15 J/cm^2^ had a statistically significant impact on SHED proliferation. Paschalidou et al. conducted a study to examine the impact of low-level laser irradiation (LLLI) at 4, 8, and 16 J/cm^2^ on the proliferation of SHEDs, along with other related factors. A statistically significant improvement in cell viability and proliferation was detected at all energy fluences examined, with the greatest impact observed between 4 and 16 J/cm^2^ [32]. Shamel et al., investigated the impact of a diode laser with a frequency of 980 nm, delivered as a single dose for 60 s, on the vitality, migration, and differentiation capacity of stem cells derived from different dental sources. Their results demonstrated enhanced cell viability among all irradiated stem cell over a period of 6 days [9] which was in agreement with our results. Gholami et al. studied the effect of photobiomodulation with 940-nm diode laser with an energy density of 4 J cm^2^ in a 100 mW continuous wave on periodontal ligament stem cells [13, 33]. In contrast to our results, they reported no difference between the proliferation levels of the laser irradiated cells and controls. However, the same group conducted a comparison between a 940 nm diode and a 660 nm red laser. Their results demonstrated that groups receiving 940 nm irradiation had superior cell proliferation and differentiation on day 3, as well as after 3 weeks of laser application. Studies on the effects of diode laser irradiation at 808 nm on mesenchymal stem cells have also recently been reported. Feng et al. stated that the migration of human gingival mesenchymal stem cells (HGMSCs) was stimulated by irradiation at 0.5–4.0 J/cm^2^, yet it did not affect proliferation [10]. Another study reported that utilizing a diode laser with a wavelength of 940 nm, as opposed to 810 nm, resulted in enhanced proliferation of periodontal ligament stem cells [34], consistent with our results and previous reports [7, 34, 35]. This difference in results may be attributed to the discrepancy in the number of irradiation sessions and the irradiation protocol. This suggests that the way laser is applied plays an important role in how well it works.

To further elucidate the impact of laser irradiation on the proliferation of human periodontal ligament stem cells (hPDLSCs), we conducted cell cycle analysis. Our results demonstrated that the application of infrared diode laser increased the percentage of hPDLSCs in the S phase and decreased the percentage of cells in the G1 phase in comparison to the control. We further investigated the effect of diode lasers on the stemness of hPDLSCs after laser irradiation. The expression of stem cell markers (*OCT*4, *SOX*2, and *NANOG*) in hPDLSCs was significantly increased following exposure to infrared diode laser irradiation. This increase in the expression of stemness markers verified the enhanced viability and proliferation of the irradiated cells in comparison to those of the nonirradiated cells. The findings of our investigation were consistent with those of Ferreira et al., who investigated the effects of subjecting SHEDs to a 660 nm diode laser at a dosage of 5 J/cm^2^ [21]. Their findings demonstrated that the application of a laser resulted in enhanced cell proliferation and the upregulation of specific markers linked to mesenchymal stem cells, including *OCT*4 and CD90. However, additional investigations into the signaling pathways involved in the enhancement of stem cell attributes in hPDLSCs by lasers are necessary to gain a more comprehensive understanding of the underlying mechanism of regeneration following infection or injury.

Next, we evaluated the impact of infrared diode laser irradiation on the osteogenic differentiation potential of hPDLSCs. RT‒PCR analysis revealed a significant upregulation of osteogenic genes in irradiated hPDLSCs compared with unirradiated cells. We examined the mRNA expressions of *RUNX2*, *ALP*, *OPN* and *OCN*. In comparison to unirradiated hPDLSCs, all studied genes were significantly overexpressed, which was in agreement with previous reports [5, 31–33]. *ALP* is considered one of the initial indicators of bone formation [36–38]. Our results demonstrated significant upregulation of *ALP* expression after 14 days of induction compared to that in nonirradiated cells. Similarly, our results demonstrated significant upregulation of *RUNX2* expression. *RUNX2* is a crucial transcription factor that is involved in skeletal development. It induces the production of several important target proteins, including *ALP*, in a highly specific and coordinated manner. Elevated levels of expression result in differentiation into the osteogenic lineage. In agreement with previous reports, we also determined that the levels of *OCN* in laser-irradiated hPDLSCs were significantly greater than those in control hPDLSCs [10, 39]. Taken together, our osteogenic gene expression results illustrated that the addition of infrared diode laser irradiation as an adjunct to the standard osteogenic induction protocol has a clear additive effect.

The regenerative potential of hPDLSCs can be enhanced by comprehending and manipulating the cell cycle phases. Consequently, we conducted cell cycle analysis to ascertain the extent to which laser irradiation facilitated the transition between various cell cycle phases, thereby promoting cell growth and specialization. Our results demonstrated that infrared diode laser irradiation enhanced the percentage of hPDLSCs in the S phase and reduced the percentage of hPDLSCs in the G1 phase in comparison to the control. This may be explained by the fact that laser irradiation operates by emitting low-intensity laser light that penetrates the cells and stimulates a variety of cellular processes which expedites the synthesis of DNA and the division of cells by facilitating the transition of cells from the G1 phase to the S phase [40]. Consequently, hPDLSCs that were exposed to infra-red diode laser demonstrated enhanced regenerative capabilities, rendering this approach a valuable asset in the fields of regenerative medicine and dental tissue engineering.

Finally, the present investigation faced some limitations since it was carried out using only one wavelength. Additional research is necessary to investigate various laser parameters and durations of irradiation to determine the optimal procedure for inducing osteogenic differentiation of hPDLSCs. Furthermore, additional research on other types of stem cells is necessary to ultimately optimize a comprehensive set of guidelines for this objective. Furthermore, it is necessary to carry out further in vivo experimental trials using irradiation parameters comparable to those used in this study to develop thoroughly studied clinical protocols and finally ascertain the most efficient therapy approach in the field of periodontal regeneration.

It is thus important to point out that standardizing experimental conditions is crucial for achieving consistent and reproducible results in research on the impact of laser irradiation on cell proliferation and differentiation. It is recommended to ensure uniformity in laser parameters, including wavelength, energy density, irradiation time, and mode of delivery. Consistency in the source of cells used, along with standardized cell culture conditions such as culture medium, growth factors, and cell density, is also essential. By implementing these strategies, more consistent and reliable results, advancing the field of laser therapy in regenerative medicine can be achieved.

In conclusion, infrared diode laser irradiation of hPDLSCs at a wavelength of 970 nm and under the irradiation conditions used in this study effectively promoted cell proliferation and enhanced their osteogenic differentiation potential. Finally, and although the integration of infrared diode laser with hPDLSCs has the potential to promote periodontal regeneration, additional clinical investigations are necessary to verify these findings and to establish optimized protocols.

## Data Availability

No datasets were generated or analysed during the current study.

## Abbreviations

LLLT: Low-level laser therapy
hPDLSCs: human periodontal ligament stem cells
DMEM: Dulbecco’s modified Eagle’s medium
PBS: Phosphate buffered solution
MSCs: Mesenchymal stem cells
MTT assay: 3-[4,5-dimethylthiazol-2-yl]-2,5 diphenyl tetrazolium bromide
Oct4: POU class 5 homeobox
Sox2: SRY-box transcription factor 2
Nanog: Nanog homeobox
Runx2: Runt-related transcription factor 2
ALP: Alkaline phosphatase
OPN: Osteopontin
OCN: Osteocalcin
